# Reduced ribosomes of the apicoplast and mitochondrion of *Plasmodium* spp. and predicted interactions with antibiotics

**DOI:** 10.1098/rsob.140045

**Published:** 2014-05-21

**Authors:** Ankit Gupta, Priyanka Shah, Afreen Haider, Kirti Gupta, Mohammad Imran Siddiqi, Stuart A. Ralph, Saman Habib

**Affiliations:** 1 Division of Molecular and Structural Biology, CSIR-Central Drug Research Institute, Lucknow, India; 2 Department of Biochemistry and Molecular Biology, Bio21 Molecular Science and Biotechnology Institute, The University of Melbourne, Victoria 3010, Australia

**Keywords:** ribosomes, Apicomplexa, organelles, large subunit (LSU) proteins, small subunit (SSU) proteins, antibiotics

## Abstract

Apicomplexan protists such as *Plasmodium* and *Toxoplasma* contain a mitochondrion and a relic plastid (apicoplast) that are sites of protein translation. Although there is emerging interest in the partitioning and function of translation factors that participate in apicoplast and mitochondrial peptide synthesis, the composition of organellar ribosomes remains to be elucidated. We carried out an analysis of the complement of core ribosomal protein subunits that are encoded by either the parasite organellar or nuclear genomes, accompanied by a survey of ribosome assembly factors for the apicoplast and mitochondrion. A cross-species comparison with other apicomplexan, algal and diatom species revealed compositional differences in apicomplexan organelle ribosomes and identified considerable reduction and divergence with ribosomes of bacteria or characterized organelle ribosomes from other organisms. We assembled structural models of sections of *Plasmodium falciparum* organellar ribosomes and predicted interactions with translation inhibitory antibiotics. Differences in predicted drug–ribosome interactions with some of the modelled structures suggested specificity of inhibition between the apicoplast and mitochondrion. Our results indicate that *Plasmodium* and *Toxoplasma* organellar ribosomes have a unique composition, resulting from the loss of several large and small subunit proteins accompanied by significant sequence and size divergences in parasite orthologues of ribosomal proteins.

## 2. Introduction

*Plasmodium* parasites have three genomes [1]: a 23 Mb nuclear genome distributed on 14 linear chromosomes [2], a 35 kb circular genome found in the relic plastid (the apicoplast) [3] and a 6 kb linear genome in the mitochondrion [4,5]. Each of these genomes is transcribed by its own apparatus [6–8] and each compartment possesses a suite of unique ribosomes for its translation [9–11]. Recent reports have provided insights into the partitioning, function and antibiotic interactions of organellar translation factors in *Plasmodium* spp. [12–16].

Eukaryotic ribosomes consist of one large (60S) and one small (40S) subunit which come together during translation to form an 80S particle. By contrast, ribosomes of bacterial origin consist of a large (50S) and small (30S) subunit that assemble to form a 70S particle. Consistent with their endosymbiotic origins, the apicoplast and mitochondria contain 70S ribosomes that are distinguishable in size (around 20 nm) from the 80S eukaryotic-type ribosomes (around 25–30 nm) found in the cytosol and rough endoplasmic reticulum (ER) [9,17,18]. In addition to ultrastructural characterization, early sequencing of organellar DNA revealed bacterial-type rRNA molecules on the mitochondrial and apicoplast genomes [19,20], although the unexpected presence of the apicoplast understandably gave rise to confusion between apicoplast and mitochondrial DNA in some of the earliest analyses [21]. Further sequencing of the 35 kb apicoplast genome revealed the presence of a complete set of rRNAs as well as a cluster of ribosomal proteins of clear plastid and bacterial origins [3]. Complete sequencing of the 6 kb mitochondrial genome revealed a collection of fragmented rRNA molecules, but no ribosomal proteins [19,22].

Initial analysis of sequenced *Plasmodium* nuclear DNA fragments and expressed sequence tags (ESTs), then later assembly of the entire *Plasmodium falciparum* nuclear genome, revealed many more ribosomal proteins with apicoplast and mitochondrial targeting sequences [2,23] that are post-translationally processed for targeting to organelles. The subsequent sequencing of organellar and nuclear genomes from a large number of other apicomplexans has expanded our picture of ribosomal and other translation components in organelles. Here, we attempt to clarify the complement of the core protein translation components by performing a cross-species survey of ribosomal proteins and ribosome assembly factors required for organellar translation in apicomplexans.

Our survey identifies considerable divergence between the organellar ribosomes of apicomplexan parasites and the ribosomes characterized in bacteria or other endosymbiotic organelles. In addition to very significant sequence and size divergences in identified orthologues of ribosomal proteins, several ribosomal proteins are either missing or sufficiently divergent to be unrecognizable. Within the phylum, we also detect several differences in ribosomal protein composition, both in those encoded by apicoplast genomes and those found in the nucleus.

Using the conserved ribosomal proteins and rRNA species identified, we have assembled structural models of the sections of the apicoplast and mitochondrial ribosomes to predict interactions of those ribosomes with parasite-killing drugs predicted to bind to bacterial ribosomes. We find considerable differences in these predicted drug–ligand interactions, with several of the modelled structures suggesting specificity of inhibition between apicoplast and mitochondrial ribosomes.

## 3. Results and discussion

### 3.1. Compositional analysis of apicoplast and mitochondrial ribosomes of *Plasmodium falciparum*

We conducted a survey of available sequences of apicomplexan apicoplast genomes, comparing ribosomal proteins encoded by different species. A list of ribosomal proteins was first assembled, based particularly on the well-annotated nuclear and organellar genomes of the red alga *Cyanidioschyzon merolae* [24–26] and the diatom *Thalassiosira pseudonana* [27,28]. We used several search strategies—genome projects were interrogated by text searches to find all annotated ribosomal proteins, and these were manually examined, gene models and predicted proteins were subject to blastp searches, whereas genome nucleotide data were subjected to tblastn searches. The OrthoMCL database of orthology groups [29] was also searched to find relevant homologues of ribosomal proteins.

### 3.2. Organellar genomes

These searches revealed several ribosomal proteins on the apicomplexan organellar genomes that had previously been missed as open reading frames (ORFs), or annotated as hypothetical ORFs. The 50S ribosomal protein L11 had previously been annotated on the *Toxoplasma gondii* apicoplast genome [30] but the syntenic protein on the *P. falciparum* genome had been hitherto annotated as orf129 [3]. This protein can now be assigned as the missing 50S L11 (table 1; gene IDs detailed in the electronic supplementary material, table S1). Similarly, the apicoplast 30S ribosomal proteins S4, S7 and S19 and the 50S proteins L4 and L36 had previously not been annotated in *Babesia—*we found ORFs on the *B. bovis* apicoplast genome that correspond to S19 and L36 at similar positions as on the *P. falciparum* apicoplast genome (table 1; electronic supplementary material, table S1).

**Table 1.** Plastid and mitochondrial ribosome large subunit (LSU) and small subunit (SSU) proteins identified for apicomplexan parasites (*P. falciparum*, *T. gondii*, *B. bovis*, *T. parva* and *E. tenella*), red alga (*C. merolae*), green alga (*C. reinhardtii*) and diatom (*T. pseudonana*). #, assigned by sequence similarity or by excluding other organellar counterpart, but targeting leader is non-obvious; $, contains an internal stop codon that may be suppressed. The L7–L12 dimer in eukaryotes is referred to as L8, but L7 and L12 are represented by a single gene in bacteria and organelles. Ticks in *black* correspond to nuclear-encoded proteins, ticks in *red* correspond to mitochondrial-encoded proteins and ticks in *green* correspond to plastid-encoded proteins. Crosses on grey background correspond to proteins for which a comprehensive search was performed on organellar and nuclear genomes and failed to detect any orthologue. Only plastid-encoded ribosomal proteins are listed for the Apicomplexans *Babesia*, *Theileria* (Piroplasmida) and *Eimeria* (Coccidia).

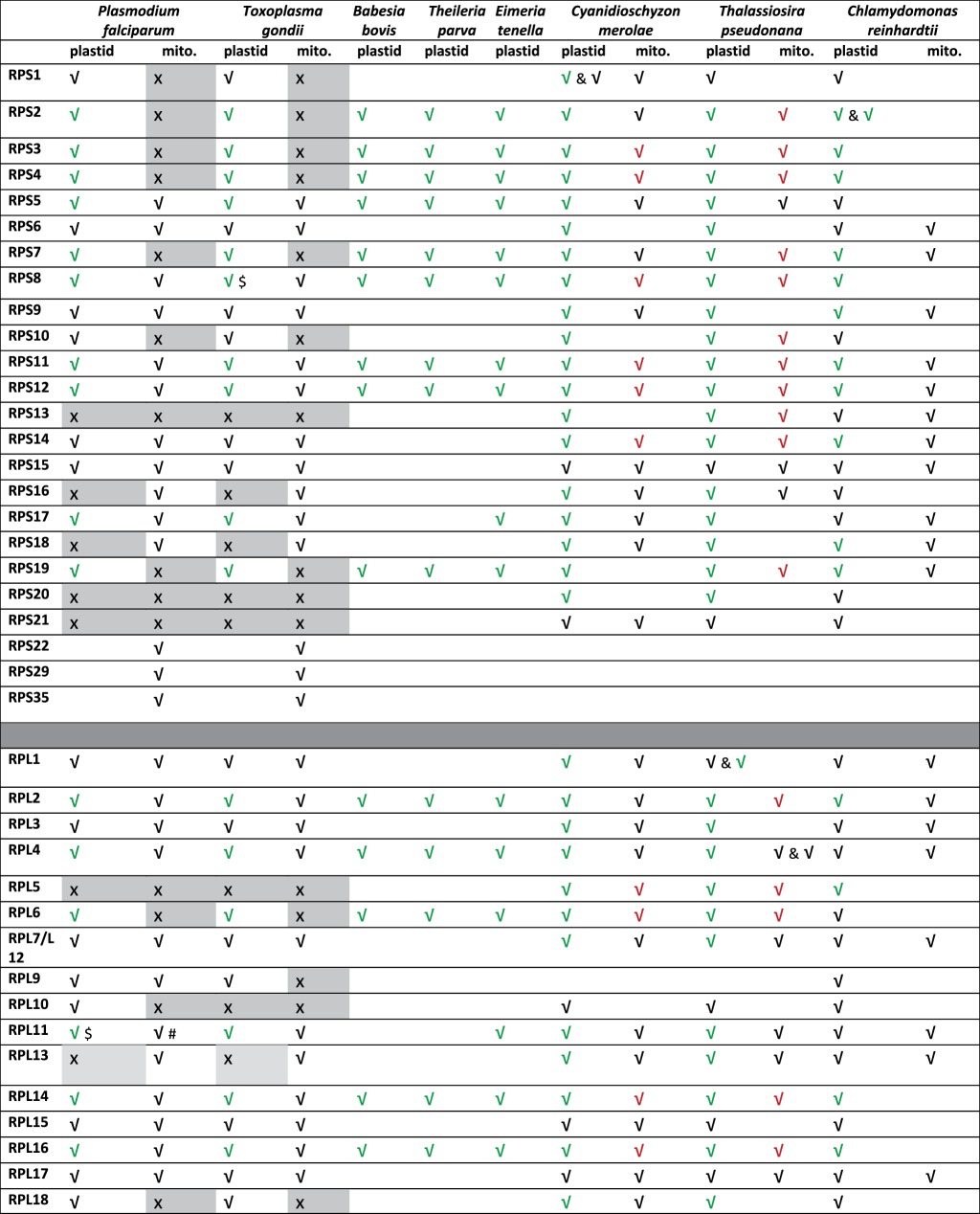
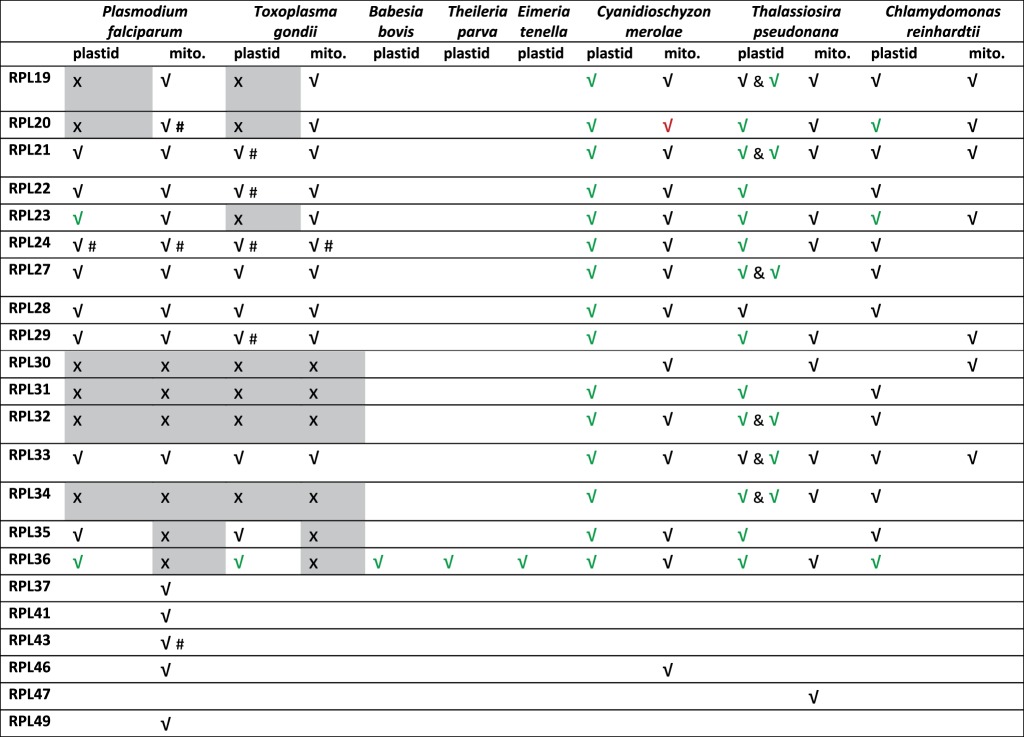

Several differences are seen between the apicoplast ribosomal complements of apicomplexan parasites. Of those proteins encoded by the apicoplast genome itself, the 50S protein L23 is present in *Plasmodium*, but absent from the *Toxoplasma* apicoplast genome, and undetectable in its nuclear genome. Rpl23 has also been previously noted as missing from the *Eimeria* apicoplast genome [31] and is not apparent in other apicomplexan genomes (table 1). This protein, thought to be involved in chaperone docking, is non-essential for growth in *Bacillus subtilis* [32], and eukaryotes, eubacteria and archaea have divergent ribosomal structures around the L23 site [33] so its absence in some apicomplexan parasites is plausible. Some chloroplast genomes lack L23; and in spinach, the role of L23 has been postulated to be replaced by chloroplast targeting of a eukaryotic 60S type L23a/L25 [34]. However, no N-terminal targeting sequences are apparent on the corresponding *Toxoplasma* genes.

Another difference between apicoplast genomes within Apicomplexa is the presence or absence of the ribosomal protein S17 (table 1). *Plasmodium*, *Toxoplasma* and *Eimeria* apicoplast genomes carry this gene, but it appears to have been lost from the apicoplast genomes of the piroplasm parasites *Theileria* and *Babesia.* We found no evidence for transfer of this apicoplast gene to the nucleus in these parasites (though mitochondrial S17 representatives are present), but S17 is small and relatively poorly conserved at a primary sequence level, so may simply be undetectable in the order Piroplasmida.

### 3.3. Missing large subunit ribosomal proteins

A number of large subunit (LSU) organellar ribosomal proteins appear to have been lost altogether from apicomplexan genomes. A striking apparent absence in Apicomplexa is the organellar Rpl5. L5 is a 5S rRNA-binding protein that is essential for assembly of the 50S central protuberance in bacteria [35], most of which is clearly retained in apicomplexan ribosomes; however, L5 is missing from mammalian mitochondrial ribosomes [36], so is a plausible absence from apicomplexan organellar ribosomes as well. Another 50S ribosomal protein that binds the 5S rRNA, L25, is missing from other plastid and mitochondrial ribosomes [36,37] and is also absent from apicomplexan genomes.

The 50S ribosomal protein Rpl10 is an apicoplast-targeted protein readily detected in *Plasmodium*, *Babesia and Theileria*, but not in *Toxoplasma.* L10 is relatively large and well conserved with clear apicoplast-targeted orthologues in *Plasmodium* spp. but no equivalent is obvious in *Toxoplasma*. Mitochondrial L10 homologues are not obvious for any apicomplexan species. In some other organelles, L10 appears to have been replaced by a nuclear L10 [38], but no L10 with an N-terminal targeting sequence is apparent in *Toxoplasma*.

**Figure 1.**
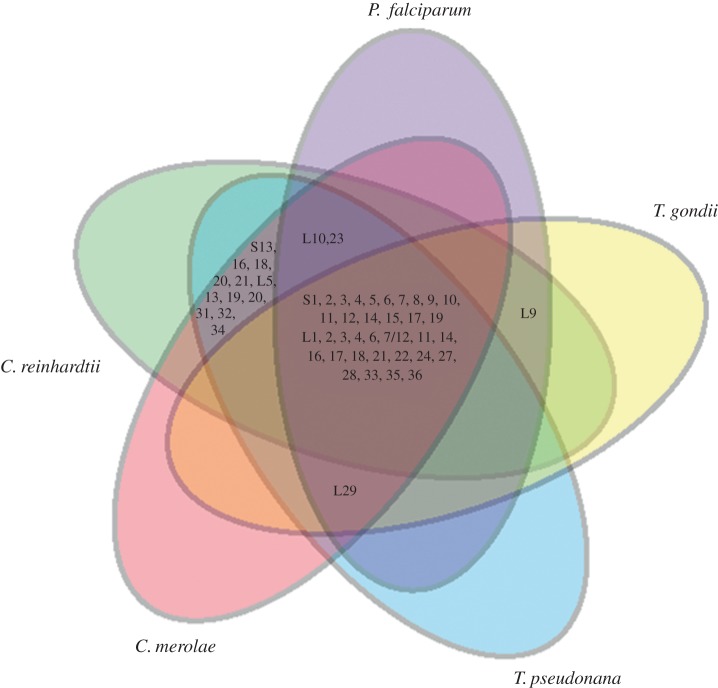
A five-set Venn diagram showing the distribution of nuclear- or plastid-encoded ribosomal proteins that would constitute the plastid ribosomes of apicomplexans *P. falciparum* and *T. gondii*, red alga *C. merolae*, green alga *C. reinhardtii* and diatom *T. pseudonana*.

The L19 and L20 proteins have orthologues in *Plasmodium* and *Toxoplasma* with probable mitochondrial targeting sequences, but we found no orthologues of these with apicoplast targeting leaders (table 1 and figure 1). These are small proteins (approx. 120 amino acids each) and may hence be missed in a sequence similarity search. Several other 50S ribosomal proteins—L30, L31, L32 and L34—have mixed distributions in other organellar ribosomes [37,39,40], and we found no apicoplast or mitochondrial representatives of any of these proteins in apicomplexans.

### 3.4. Missing small subunit ribosomal proteins

Compared to the 50S subunit, the 30S subunit retains proportionally more members in the apicoplast genome rather than transfers to the nuclear genome (table 1; gene IDs detailed in the electronic supplementary material, table S1). Several proteins are also missing or undetected among the 30S proteins of the mitochondria and apicoplast. The mitochondrion in particular appears to be missing a large number of subunits, and we were unable to find mitochondrial targeted orthologues of S1, S2, S3, S4, S7, S10, S13, S19, S20 or S21. Most of these are retained on the mitochondrial genomes of diatoms (table 1) and are widely conserved among other organellar ribosomes, so their complete absence in apicomplexan mitochondria is unexpected and not easily explained. One possibility is that the mitochondrial ribosomes employ prokaryotic subunits encoded by the apicoplast (though no mechanism is obvious for this) or may use proteins dually targeted to the mitochondrion and apicoplast. Several apparently mitochondrial targeted proteins are annotated as 30S ribosomal proteins in apicomplexans, including S22, S29 and S35. These are not widespread members of mitochondrial ribosomes, so their presence here may be linked to the possible absence of other canonical members.

Several 30S proteins are also apparently lacking in the apicoplast ribosomes. Despite the presence of clear orthologues in red algal and diatom organelles (table 1), no apicoplast (or mitochondrial) S13 ribosomal proteins are apparent in any apicomplexan species. This protein interacts with the 50S subunit and the P-site tRNA during translocation [41] and is essential for translation in other bacterial ribosomes [42], so its apparent absence is puzzling [42].

Apicomplexan parasites also appear to have lost their apicoplast version of S16, which is encoded on the plastid genomes of diatoms and of red and green alga, but have retained mitochondrial targeted S16 proteins (table 1 and figure 1). In bacteria, S16 is essential and plays a central role in 30S ribosomal assembly [43]. S16 is dual targeted between mitochondria and chloroplasts in many plants [44] but we see no evidence for the presence of a possible apicoplast leader upstream of the mitochondrial S16 in *Plasmodium* or *Toxoplasma*. Another 30S protein, S18 is absent from all the apicomplexan apicoplasts we surveyed, though mitochondrial S18 s are present (table 1). This protein has no obvious orthologue in archaeal or eukaryotic ribosomes [45], although it is essential in tobacco plastids [46].

S20 and S21 are also missing from the apicomplexans we surveyed. S20 is not essential in *Salmonella* [47], knockout of S21 impairs but does not ablate translation in other plastids [48], and neither is essential in *B. subtilis* [32] so these are plausible absences from the organellar ribosomes of Apicomplexa.

### 3.5. Ribosome assembly proteins for the apicoplast and mitochondrion

Ribosome biogenesis involves multiple steps of ribosomal RNA (rRNA) processing and association of rRNA with ribosomal proteins [49–51]. As with any complex RNA molecule, the rRNA in parasite organelles is prone to the formation of numerous local non-native secondary structures. A set of cofactors known as ribosomal biogenesis/assembly factors prevents formation of these stable, misfolded regions in the rRNA and promotes ribosome assembly [50]. These factors serve as check points during the assembly process where they mediate proper rRNA folding and protein–RNA interactions by creating specific nucleotide modifications in rRNA or by acting as RNA/protein chaperones. This ultimately results in the assembly of mature ribosomal subunits. We performed an extensive search for putative ribosomal biogenesis factors targeted to the apicoplast and mitochondrion in the PlasmoDB genome database using current annotations as well as new assignments based on targeting prediction algorithms.

Ribosome assembly factors belong to the following broad categories—GTPases, chaperones/maturation factors and DEAD-box proteins [52]. GTP hydrolysis by proteins of the GTPase superclass is involved at different stages of ribosome biogenesis mediating subunit assembly. Era, Der, Obg and YihA are known to interact with either the mature subunits or the 70S ribosome while YlqF also exhibits interaction with a ribosomal subunit intermediate [53]. Sequence analysis indicates the presence of multiple P-loop GTPases in *P. falciparum* that contain highly conserved motifs (table 2). The Der protein is conserved among eubacteria but not in archaea or eukaryotes [52]; two Der homologues, with predicted targeting to the apicoplast and mitochondrion, respectively, could be identified. A search for homologues of organellar Era and YihA proteins yielded putative candidates with mitochondrial localization while the single YlqF homologue had apicoplast targeting elements. Two candidates were found for Obg, one of which was predicted to be mitochondrial while the other appears to be targeted to the apicoplast.

**Table 2.** Organellar ribosome assembly proteins of *P. falciparum* and their predicted targeting.

s. no.	ribosome assembly proteins	putative interactions and functions	PlasmoDB annotation	PlasmoDB gene ID	apicoplast targeting			mitochondrial targeting		probable organellar destination
					PlasmoAP	PATS	signal peptide (TargetP)	PlasMit	MitoProt II	
GTPases										
1	Era/Bex	involvement in 16S rRNA processing and 30S subunit biogenesis	GTPase, putative	PF3D7_1435800	−/++	no (0.048)	no (0.024)	mito. (91%)	(0.550)	mitochondrion
2	Der/EngA/YfgK/YphC	association with 50S subunit and involvement in its maturation	GTP-binding protein, putative	PF3D7_1217300	0/++	yes (0.955)	yes (0.929)	non-mito. (99%)	(0.570)	apicoplast
			GTP-binding protein EngA, putative	PF3D7_0313500	−/++	no (0.062)	no (0.051)	mito. (91%)	(0.409)	mitochondrion
3	Obg/CgtA_E_/YhbZ/ObgE	association with 30S and 50S subunit; also co-sediments with 16S and 25S rRNA	GTP-binding protein, putative	PF3D7_1411600	+/++	yes (0.970)	yes (0.806)	non-mito. (99%)	(0.860)	apicoplast
			GTP-binding protein, putative	PF3D7_0824300	−/++	no (0.392)	no (0.037)	mito. (91%)	(0.924)	mitochondrion
4	YihA/EngB/YsxC	interaction with Der protein and activation of its GTP activity. Involvement in 50S subunit assembly	GTP-binding protein, putative	PF3D7_0513400	−/++	no (0.058)	no (0.030)	mito. (91%)	(0.842)	mitochondrion
			GTP-binding protein, putative	PF3D7_1442200	−/++	no (0.061)	no (0.029)	mito. (91%)	(0.674)	mitochondrion
5	YlqF/RbgA	involvement in 50S subunit assembly; co-sediments with 45S intermediate	GTPase, putative	PF3D7_0410700	++/++	yes (0.976)	yes (0.928)	non-mito. (99%)	(0.526)	apicoplast
maturation factors and chaperones										
6	RimM	interaction with RP-S19 in the free 30S subunit and involvement in 16S rRNA processing	mitochondrial preribosomal assembly protein rimM precursor, putative	PF3D7_1032000	++/++	yes (0.992)	yes (0.971)	non-mito. (99%)	(0.560)	apicoplast
7	RlmE/RrmJ/FtsJ	specific methylation at uridine of 23S rRNA in the fully assembled 50S subunit	rRNA methyltransferase, putative	PF3D7_1309600	−/+	no (0.451)	no (0.087)	non-mito. (99%)	(0.213)	
			large subunit rRNA methyltransferase, putative	PF3D7_1354300	−/++	no (0.022)	no (0.035)	mito. (91%)	(0.145)	mitochondrion
			rRNA methyltransferase, putative	PF3D7_0908600	−/++	no (0.023)	no (0.031)	non-mito. (99%)	(0.149)	
8	RsmB/Sun/RrmB/Fmu	specific methylation at cytosine of 16S rRNA	methyltransferase, putative	PF3D7_1020400	−/++	no (0.030)	no (0.120)	mito. (91%)	(0.525)	mitochondrion/apicoplast
9	KsgA/RsmA/Dim1	specific di-methylation at two adjacent adenosines near 3′ end of 16S rRNA in the 30S particle	small subunit rRNA dimethylase, putative	PF3D7_1415800	−/++	no (0.185)	no (0.086)	mito. (91%)	(0.981)	mitochondrion [54]
			apicoplast dimethyladenosine synthase, putative	PF3D7_1249900	++/++	yes (0.999)	yes (0.941)	non-mito. (99%)	(0.996)	apicoplast
10	DnaJ/HSP40	chaperone	heat shock protein 40 (DnaJ)	PF3D7_0409400	−/++	yes (0.900)	no (0.032)	non-mito. (99%)	(0.474)	apicoplast targeting demonstrated [55]
			DnaJ protein, putative	PF3D7_0629200	0/0	yes (0.502)	yes (0.982)	non-mito. (99%)	(0.450)	apicoplast
11	DnaK/HSP70		heat shock protein 70 (Hsp70-3)	PF3D7_1134000	−/++	no (0.046)	no (0.027)	mito. (91%)	(0.443)	mitochondrion
12	GroEL/Cpn60		heat shock protein 60 (HSP60)	PF3D7_1015600	−/++	no (0.019)	no (0.043)	mito. (91%)	(0.951)	mitochondrial targeting demonstrated [56]
			60 kDa chaperonin (CPN60)	PF3D7_1232100	++/++	yes (0.979)	yes (0.776)	non-mito. (99%)	(0.824)	apicoplast targeting demonstrated [56]
13	GroES/Cpn10		10 kDa chaperonin (CPN10)	PF3D7_1215300	−/−	no (0.317)	no (0.110)	non-mito. (99%)	(0.493)	mitochondrial targeting demonstrated [56]
14	Cpn20		20 kDa chaperonin (CPN20)	PF3D7_1333000	++/++	no (0.944)	yes (0.931)	non-mito. (99%)	(0.665)	apicoplast targeting demonstrated [56]

Chaperones assist in proper folding/unfolding and assembly/disassembly of ribosomal proteins and rRNA. We identified seven chaperones, five of which are already annotated in previous reports as being targeted to the apicoplast (DnaJ, Cpn60 and Cpn20) or mitochondrion (Cpn60 and Cpn10) [55,56]. Two other putative chaperones—DnaJ and DnaK—that might be apicoplast- and mitochondrion-targeted, respectively, were also identified (table 2). In addition to chaperones, RNA maturation factors play a vital role in the rRNA modifications during ribosome biogenesis. RrmJ, RsmB and KsgA are methyltransferases that methylate specific nucleotides in rRNA during their maturation [57–59]. KsgA methylates two adjacent adenosine residues at the 3′ terminal helix of small subunit (SSU) rRNA that are two of three nucleotide modifications that are known to be conserved in nearly all known ribosomes throughout evolution [60] with few exceptions [61–64]. Two homologues of RlmE/RrmJ were identified in the *P. falciparum* genome, one of which had a predicted mitochondrial targeting signal while the location of the other cannot be clearly predicted. Two KsgA/RsmA were predicted, one for the mitochondrion and the other with possible dual targeting to both organelles. A single RsmB with possible dual targeting to the apicoplast and mitochondrion was also identified. Further, a homologue of the ribosomal maturation factor RimM that is involved in SSU biogenesis [65] is predicted for the apicoplast (table 2).

DEAD-box proteins, which are conserved across bacteria and viruses to humans [66], belong to a large family of RNA helicases that possess RNA-dependent ATPase activity. They act as RNA chaperones, mediate RNA–protein interaction and unwind local RNA structures [67–69]. A number of putative proteins that may belong to the DEAD-box family and are predicted to contain sequence elements for organellar import (PF3D7_1445900, PF3D7_0218400, PF3D7_1332700, PF3D7_1418900, PF3D7_0504200, PF3D7_1021500 and PF3D7_1251500) were identified. These proteins have a conserved DEAD-box motif and RNA helicase domain but could not be unambiguously classified as a specific member (SrmB, CsdA, DbpA, RhlE or RhlB) of the *Escherichia coli* DEAD-box helicase family [70–74].

### 3.6. Structure modelling of *Plasmodium falciparum* organellar ribosome subunits and drug interaction sites

Several antibiotics, including clindamycin, chloramphenicol and the macrolides erythromycin and azithromycin, bind in the vicinity of the ribosome LSU peptidyl transferase centre or the peptide exit tunnel and inhibit parasite growth. This group also includes thiostrepton that contacts ribosomal protein L11 and the GTPase region of 23S rRNA [75]. Translation inhibitory antibiotics have two putative target organelles, the apicoplast and mitochondrion, of the parasite. Some antibiotics (e. g. clindamycin, azithromycin, chloramphenicol and tetracycline) have been demonstrated to have a delayed-death effect, a phenotype associated with apicoplast-specific action [76,77]. A single point mutation in the LSU rRNA gene of the *T. gondii* apicoplast confers clindamycin resistance *in vitro* [78] and resistance to azithromycin in *P. falciparum* has been attributed to two point mutations: one in the *P. falciparum* apicoplast LSU rRNA and a second in the apicoplast-encoded Rpl4 [79]. Thiostrepton causes immediate parasite killing and is proposed to have additional targets in *P. falciparum* [80,81]. In order to understand the differential interaction of these drugs with apicoplast and mitochondrial ribosomes, we carried out *in silico* modelling of LSU rRNA and relevant ribosomal proteins (L4, L11 and L22) involved in interactions with antibiotics in bacteria. This was followed by docking of antibiotics in order to estimate their relative specificity for mitochondrial and apicoplast ribosomes.

**Figure 2.**
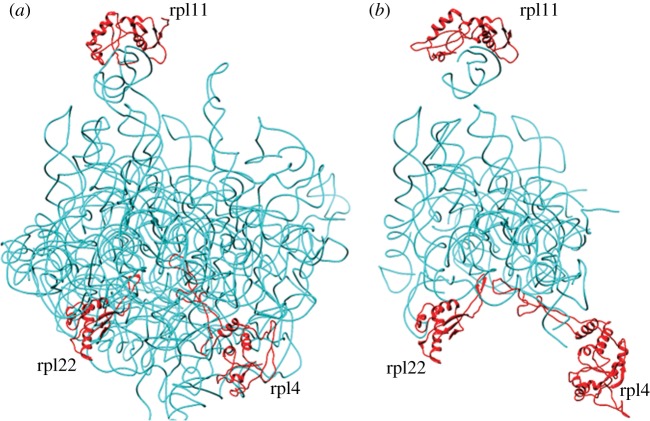
Structure models of *P. falciparum* apicoplast (*a*) and mitochondrial (*b*) LSU rRNA and proteins L11, L4 and L22. The rRNA and protein subunits were modelled separately and superimposed on the *E. coli* ribosome template to generate the ribosome complexes. LSU rRNA is shown in cyan and proteins in red.

Prediction of the three-dimensional structures of parasite organelle ribosomes is demanding due to the difficulty in obtaining high-resolution experimental models. This is further complicated by the presence of highly fragmented rRNA encoded by the *P. falciparum* mitochondrial genome [11]. A stand-alone version of the RNA prediction tool ModeRNA was used for the comparative modelling of rRNA, whereas modelling of ribosomal proteins L4, L11 and L22 was performed by Modeller v. 9.10. For modelling of the mitochondrial ribosome, different fragments of mitochondrial LSU rRNA were aligned manually on the basis of conserved secondary structure topology, modelled separately and then superimposed together on the *E. coli* template to obtain a complex RNA model structure. All the modelled subunits (rRNA and protein) were superimposed on the *E. coli* ribosome template to generate the apicoplast and mitochondrial ribosome complexes (figure 2). The fragmented rRNA comprising the core of the mitochondrial ribosome is highly reduced, though retains conservation of the peptidyl transferase centre and the peptide exit tunnel where most antibiotics bind.

**Figure 3.**
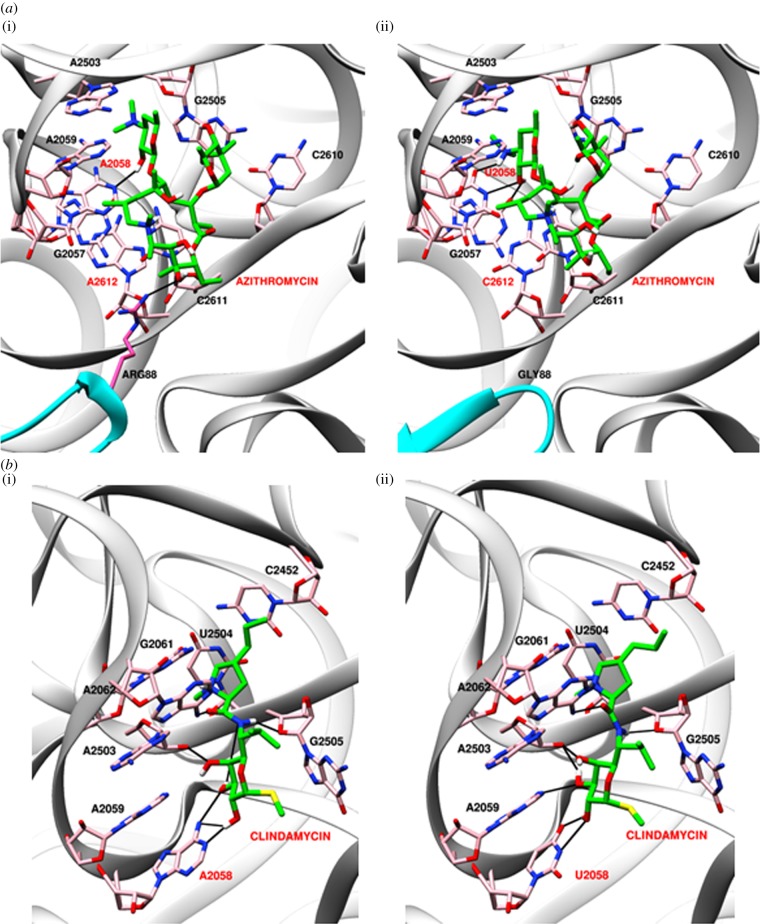
Modelling of antibiotic interactions with *P. falciparum* organelle ribosomes. (*a*) Azithromycin docked onto apicoplast (i) and mitochondrial (ii) ribosomes. As in the *Thermus thermophilus* ribosome–azithromycin structure, a single azithromycin molecule was docked at the binding site. (*b*) Interaction of clindamycin with apicoplast (i) and mitochondrial (ii) LSU rRNA. Bases that differ between the apicoplast and mitochondrial rRNA are shown in red and H-bonds as black lines. rRNA is in grey, L22 in cyan and antibiotics are in green.

Molecular docking of antibiotics was performed on *P. falciparum* apicoplast and mitochondrial ribosome models using Autodock4. Autodock uses grid-based energy evaluation for docking, where ligands are treated as flexible entities by exploring torsional degrees of freedom of ligand molecules. The first step of the Autodock algorithm involves conformational sampling of ligands followed by prediction and ranking of free energy of binding of these conformations. One hundred Autodock runs were performed for each inhibitor. To validate the reproducibility and sensitivity of the docking program, Autodock4 was used to dock the inhibitor co-complexed with the *E. coli* template. The inhibitor dock scores obtained for apicoplast and mitochondrial ribosomes are given in table 3. In the apicoplast, L22 located at the binding site for azithromycin [82] contains Arg88 that is predicted to form an H-bond with the inhibitor (figure 3*a*). Arg88 is replaced by Gly88 in mitochondrial L22 that does not form an H-bond with azithromycin. In addition, the rRNA sequence at the binding site also differs at two positions: A2612 and A2058 (*E. coli* number) in the apicoplast are replaced by C2612 and U2058, respectively, in the mitochondrion, a change that would alter the hydrophobic environment at the site. This might explain the differential specificity of azithromycin for organellar ribosomes. The higher affinity of the antibiotic for the apicoplast ribosome is also reflected in the lower dock scores obtained for azithromycin and the related macrolide erythromycin (table 3). Together with the LSU rRNA, L22 and L4 are predicted to form the peptide exit tunnel on the ribosome. The G76V mutation of apicoplast L4 has been reported to contribute to azithromycin resistance in *P. falciparum* lines [79] and modelling on the ribosome–azithromycin structure predicted a conformational shift in the side chain of Leu75 of L4 that could interfere with the azithromycin binding pocket. However, this model was constructed on the *Deinococcus radiodurans* (an extremophile bacterium) model that proposed the binding of two azithromycin residues at the site, one that interacted with the LSU rRNA and the other with L4, L22 and LSU rRNA [83]. Structures of the *Haloarcula marismortui* (an archaeon) and *Thermus thermophilus* large ribosomal subunits complexed with azithromycin have since led to the conclusion that a single molecule of the antibiotic binds to the ribosome [82]. This is supported by biochemical experiments that indicate that only one azithromycin molecule is bound to the *E. coli* ribosome [84]. No direct role for L4 in the interaction of azithromycin with *P. falciparum* apicoplast and mitochondrial ribosomes was detected in our model.

**Table 3.** Docking scores of antimicrobials in the active site of large ribosomal subunit of *E. coli*, and *P. falciparum* apicoplast and mitochondrion.

	antibiotic	*P. falciparum* apicoplast LSU		*P. falciparum* mitochondrial LSU		*E. coli* LSU	
		dock score (kcal mol^−1^)	rmsd (Å)	dock score (kcal mol^−1^)	rmsd (Å)	dock score (kcal mol^−1^)	rmsd (Å)
1	chloramphenicol	−3.44	1.31	−3.19	1.33	−3.55	1.25
2	erythromycin	−13.85	1.01	−11.04	1.61	−12.2	0.97
3	azithromycin^a^	−21.47	0.7	−18.3	1.78	−18.64	0.64
4	clindamycin	−15.97	1.13	−14.44	1.57	−14.94	1.07
5	thiostrepton^b^	−2.69	3.68	−1.98	1.69	−2.31	0.68

^a^Modelled on the *Thermus thermophilus* ribosome–azithromycin crystal structure.

^b^Modelled on the *Deinococcus radiodurans* ribosome–thiostrepton crystal structure.

The only difference in the interaction site for clindamycin between the organelle ribosomes was an A2058U (*E. coli* number) transversion in the LSU rRNA of the parasite mitochondrion (figure 3*b*). This residue forms H-bonds with the antibiotic in *E. coli* [85]. The LSU rRNA residue G2061, whose mutation in the apicoplast is associated with clindamycin resistance in *T. gondii* [78] and which is critical to the transpeptidation reaction, was conserved in the LSU rRNA of both organelles in *Plasmodium*. The binding site for chloramphenicol overlaps with that of clindamycin and no obvious differences could be detected in chloramphenicol interactions predicted for apicoplast and mitochondrial ribosomes; the dock scores for chloramphenicol were also comparable for *P. falciparum* organelle ribosomes. However, the *in silico* approach used by us would have inherent weaknesses, and conclusions on actual interactions and affinity of these antibiotics for apicoplast/mitochondrial ribosomes awaits experimental validation.

**Figure 4.**
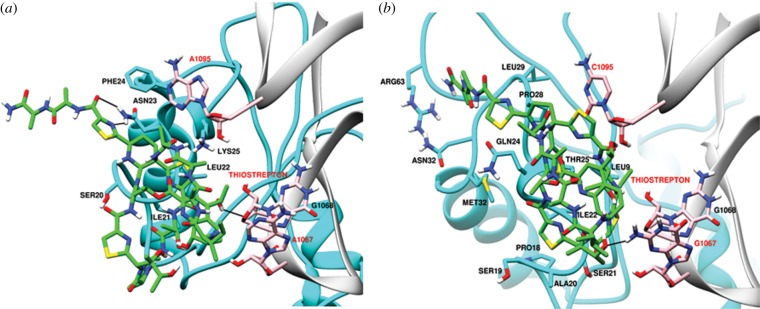
Predicted interaction of thiostrepton with LSU rRNA and L11 of ribosomes of the *P. falciparum* apicoplast (*a*) and mitochondrion (*b*). rRNA is in grey, L11 in cyan and thiostrepton in green.

**Figure 5.**
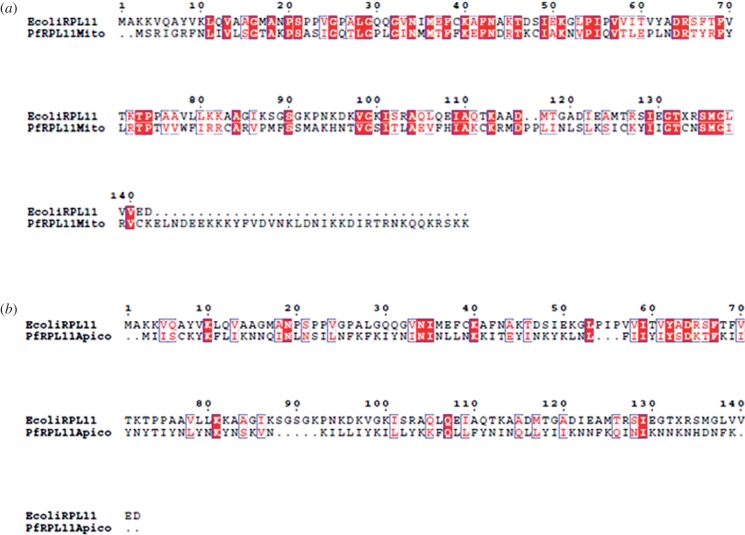
ClustalW alignment of *E. coli* L11 with L11 predicted for the *P. falciparum* mitochondrion (*a*) and apicoplast (*b*).

Thiostrepton targets the GTPase associated centre of the 50S ribosome subunit and binds within a cleft between helices 43 and 44 of the LSU rRNA and L11. It overlaps with the position of domain V of elongation factor G (EF-G), thus perturbing the binding of the elongation factor to ribosomes [86]. *Plasmodium falciparum* organelle LSU rRNAs differ at two residues in the helices: the crucial A1067 site and A1095 (*E. coli* number) are replaced by G1067 and C1095 in the mitochondrion (figure 4). The former has been shown to alter binding of thiostrepton to the ribosome although introduction of an A1067G mutation in the apicoplast rRNA did not completely abolish *in vitro* interaction with the antibiotic [87]. It is also important to note the low identity and consequent conformational differences in L11 of the apicoplast and mitochondrion that might influence interaction with thiostrepton. The structural models in figure 4 as well as the ClustalW alignment of *E. coli* and *P. falciparum* organelle L11 proteins indicate greater similarity between the bacterial and parasite mitochondrial ribosome–thiostrepton interaction site compared with the apicoplast [86] (figures 4 and 5). The identity between the mitochondrial and apicoplast L11 with the *E. coli* protein is 24.71% and 10.07%, respectively. In addition to targeting the apicoplast, thiostrepton has also been shown to act on the cytosolic proteasome [80] and has detectable effects on mitochondrial translation [81]. Thiostrepton is also able to partially lock *P. falciparum* mitochondrial EF-G onto surrogate *E. coli* ribosomes, an effect not observed with apicoplast EF-G [14].

Except for the macrolide antibiotics whose preferential interaction with *P. falciparum* apicoplast ribosomes can be explained on the basis of structural differences with ribosomes of the mitochondrion, few obvious structural explanations can be found for differential drug binding to apicoplast and mitochondrial ribosomes by other antibiotics tested by us. Apicoplast-specific inhibitory effects that have been observed with clindamycin and chloramphenicol may thus be due to differential sensitivity attributable to other biological factors such as differences in drug accumulation in the two organelles or reduced rate of translation in the parasite mitochondrion. For thiostrepton, the docking results and structural models reported here support earlier biochemical data that the antibiotic targets both apicoplast and mitochondrial translation thus mediating early parasite death.

In conclusion, apicoplast and mitochondrial ribosomes of apicomplexan parasites have a unique and reduced composition, a fact that would alter the nature of their interactions with protein translation factors. This survey is a starting point for further functional evaluation of the *Plasmodium* organellar ribosome machinery, its assembly and interactions with translation factors and translation inhibitory compounds.

## 4. Material and methods

### 4.1. Databases and sequence searches

To identify ribosomal proteins and ribosome assembly factors, we searched apicomplexan genomes using the GenBank non-redundant nucleotide and CDS translations [88] using tblastn and blastp, respectively. We additionally performed direct alignments between protein sequences and organellar genomes using blast2seq. Signal peptide portions of apicoplast targeting sequences were sought using SignalP v. 3.0 [89] and by manual inspection of Kyte Doolitle hydropathy plots [90]. Gene models were examined using EuPathDB [91] and evidence for transcription start sites and alternative splicing based on RNAseq data examined using the GBrowse tool [92] implemented at EuPathDB [91]. Putative transit peptide portions of apicoplast targeting sequence were manually inspected or were detected using the PlasmoAP [93] and PATS [94]. Putative mitochondrial transit peptides were manually inspected or were detected using PlasMit [95] or MitoProtII [96].

Where we identified clear ribosomal proteins that lacked clear annotations, or clear organellar trafficking that was not included in earlier annotations, we communicated updated annotations to curation staff. For high confidence assignments the gene names have been changed, for lower confidence assignments relevant comments have been added to gene pages at the EuPathDB [91] and GeneDB genome databases [97].

For ribosome assembly/biogenesis proteins, all predictions were made on the basis of annotations in PlasmoDB as well as assignments made by prediction algorithms—TargetP, PlasmoAP, PATS, PlasMit and MitoProt II.

### 4.2. Molecular modelling

Prediction of three-dimensional structure of ribosomal structure is highly demanding owing to the difficulty in obtaining high-resolution experimental models. Present work describes the *in silico* modelling of apicoplast and mitochondrial large subunits of 23S ribosome followed by docking studies with known inhibitors to understand the comparative basis of specificity of these inhibitors. To achieve the modelling of rRNA, an RNA prediction tool ModeRNA [98] was used, whereas modelling of ribosomal proteins L4, L11 and L22 was performed by Modeller v. 9.10 [99]. After model building of large subunit of 23S rRNA, known inhibitors azithromycin, erythromycin, clindamycin, chloramphenicol and thiostrepton were docked into the peptidyl transferase site of modelled apicoplast and mitochondrial ribosome, respectively.

ModeRNA is a comparative modelling tool of RNA which requires a template whose three-dimensional structure is known and which shares sequence similarity with the query sequence, the one to be modelled and pairwise alignment between template and the query sequences [98]. 23S ribosome of *E. coli* (PDB id: 3OFC) was chosen as the template to model *P. falciparum* 23S rRNA in apicoplast as well as in mitochondria. As the secondary structures have been published for *E. coli* and *P. falciparum* ribosomes, the alignments were performed manually on the basis of conserved secondary structure topology to facilitate the modelling of rRNA of apicoplast and mitochondria in *P. falciparum*. The RNA models were built with the stand-alone version of the ModeRNA via a Python scripting interface based on the provided alignments. The default ModeRNA modelling procedure was followed. As the *P. falciparum* mitochondrial RNA is present in fragmented form, different fragments were aligned and modelled separately and then superimposed on the template together to obtain a complex model structure. Simple geometry checks were performed using analyze_geometry function on template and target structures using ModeRNA stand-alone version to ensure the structural integrity of structure.

All the protein models were built with Modeller v. 9.10 based on homologous template structures in *E. coli*. For each case, 10 different models were produced and the one with the best DOPE score selected. ClustalW was used for alignment between protein templates and the targets to generate comparative models.

All the modelled subunits of each ribosome including RNA and protein were superimposed on template structure and merged together to form the complete ribosome. Although RNA sequences exhibit divergence, the overall structures of modelled ribosomes were found to be well conserved as indicated by the secondary structure topology.

### 4.3. Molecular docking

Structures of inhibitors were extracted from the Protein Data Bank (PDB) files of large subunit of 70S ribosome co-complexed with the respective inhibitors (PDB IDs: chloramphenicol (3OFC), clindamycin (3OFZ), erythromycin (3OFR), azithromycin (3OHZ) and thiostrepton (3CF5)). Molecular docking was performed on ribosome models used as a receptor to dock our inhibitors of interest using Autodock v. 4 [100]. Kollman charges were assigned with 40 × 40 × 40 grid points of 0.375 Å spacing. One hundred Autodock runs were performed for each inhibitor.

To validate the reproducibility and sensitivity of the docking program, Autodock v. 4 was used to dock the inhibitor co-complexed with template. The limit of Autodock to read maximum atoms of macromolecules was kept constant to default and therefore 35 Å around the ligands was considered only after superimposing the modelled structure on *E coli* ribosome structure.

## Supplementary Material

Supplementary Table 1
