# Supplementary material for: Reduced ribosomes of the apicoplast and mitochondrion of Plasmodium spp. and predicted interactions with antibiotics
Source: Open Biol. 2014 May 21;4(5):140045. doi: 10.1098/rsob.140045 (PMC4042851; doi:10.1098/rsob.140045)
Supplement: Supplementary Table 1 [file rsob140045supp1.docx]

**Supplementary Table 1**. Gene IDs for plastid and mitochondrial ribosome LSU and SSU proteins of apicomplexan parasites (*P. falciparum*, *T. gondii*, *B. bovis*, *T. parva*, *E. tenella*), red alga (*C. merolae*), green alga (*C. reinhardtii*) and diatom (*T. pseudonana*)

|  | ***Plasmodium falciparum*** | | ***Toxoplasma gondii*** | | ***Babesia bovis*** | ***Theileria parva*** | ***Eimeria tenella*** | ***Cyanidioschyzon merolae*** | | ***Thalassiosira pseudonana*** | | | ***Chlamydomonas reinhardtii*** | |
| --- | --- | --- | --- | --- | --- | --- | --- | --- | --- | --- | --- | --- | --- | --- |
|  | **Plastid** | **Mito.** | **Plastid** | **Mito.** | **Plastid** | **Plastid** | **Plastid** | **Plastid** | **Mito.** | **Plastid** | | **Mito.** | **Plastid** | **Mito.** |
| **RPS1** | PF3D7_0811900 | not found | TGME49_286620 | not found |  |  |  | BAC76171 & CMM019C | CMI304C | XP_002292331.1 | |  | EDP02480.1 |  |
| **RPS2** | CAA64575 | not found | AAD41151 | not found | YP_002290844 | XP_762713 | NP_852644 | BAC76280 | CMT330C | ABK20743 | | YP_316599 | NP_958390 & NP_958391 |  |
| **RPS3** | CAA64581 | not found | AAD41136 | not found | YP_002290872 | XP_762674 | NP_852625 | BAC76237 | NP_059375 | ABK20814 | | YP_316611 | NP_958402 |  |
| **RPS4** | CAA64576 | not found | AAD41132 | not found | YP_002290875 | XP_762670 | NP_852621 | BAC76103 | NP_059378 | ABK20802 | | YP_316600 | NP_958376 |  |
| **RPS5** | CAA64587 | PF3D7_0713600 | AAD41141 | TGGT1_037360 | YP_002290866 | XP_762682 | NP_852631 | BAC76247 | CMK274C | ABK20824 | | EED94712 | EDO99741.1 |  |
| **RPS6** | PF3D7_0932600 | PF3D7_1463800 | TGME49_017670 | TGME49_084660 |  |  |  | BAC76162 |  | ABK20839 | |  | EDP00617.1 | EDO96868 |
| **RPS7** | CAA64592 | not found | AAD41144 | not found | YP_002290863 | XP_762687 | NP_852635 | BAC76257 | CMT485C | ABK20834 | | YP_316603 | NP_958380 | XP_001690292.1 |
| **RPS8** | CAA64585 | PF3D7_0718400 | ORF at 10615-10968 of U87145.2 $ | TGME49_261380 | YP_002290868 | XP_762680 | NP_852629 | BAC76244 | NP_059370 | ABK20821 | | YP_316597 | NP_958374 |  |
| **RPS9** | PF3D7_1413400 | PF3D7_1137100 | TGME49_018850 | TGME49_207940 |  |  |  | BAC76254 | CMQ175C | ABK20831 | |  | NP_958395 | EDP07662 |
| **RPS10** | PF3D7_1460900.1 | not found | TGME49_219832 | not found |  |  |  | BAC76259 |  | ABK20836 | | YP_316596 | EDP03308.1 |  |
| **RPS11** | CAA64590 | PF3D7_1454600 | AAD41156 | TGME49_231120 | YP_002290865 | XP_762685 | NP_852633 | BAC76251 | NP_059369 | ABK20828 | | YP_316608 | NP_958411 | XP_001695507.1 |
| **RPS12** | CAA64591 | PF3D7_0412100 | AAD41143 | TGME49_219770 | YP_002290864 | XP_762686 | NP_852634 | BAC76256 | NP_059379 | ABK20833 | | YP_316602 | NP_958416 | XP_001694988.1 |
| **RPS13** | not found | not found | not found | not found |  |  |  | BAC76250 |  | ABK20827 | | YP_316595 | EDO98007.1 | EDP01270 |
| **RPS14** | PF3D7_1137500 | PF3D7_1447300 | TGME49_286610 | TGME49_277700 |  |  |  | BAC76208 | NP_059371 | ABK20757 | | YP_316617 | NP_958381 | XP_001694988.1 |
| **RPS15** | PF3D7_1106100 | PF3D7_1310900 | TGME49_116110 | TGME49_016040 |  |  |  | CMN158C | CMH066C | EED95834 | | EED94275 | XP_001696288.1 | XP_001701508.1 |
| **RPS16** | not found | PF3D7_0531200 | not found | TGME49_318420 |  |  |  | BAC76167 | CMG062C | ABK20801 | | EED94242 | EDO97973.1 |  |
| **RPS17** | CAA64583 | PF3D7_1365100 | AAD41138 | TGME49_273060 |  |  | NP_852627 | BAC76240 | CMS212C | ABK20817 | |  | EDO96943.1 | EDO98375 |
| **RPS18** | not found | PF3D7_1211500 | not found | TGME49_222990 |  |  |  | BAC76275 | CMP223C | ABK20749 | |  | NP_958392 | EDP02197 |
| **RPS19** | CAA64580 | not found | AAD41135 | not found | ORF at 26780-26541 | XP_762673 | NP_852624 | BAC76235 |  | ABK20811 | | YP_316610 | NP_958370 | EDP02706 |
| **RPS20** | not found | not found | not found | not found |  |  |  | BAC76277 |  | ABK20747 | |  | EDO98001.1 |  |
| **RPS21** | not found | not found | not found | not found |  |  |  | CMB032C | CMR201C | XP_002286470 | |  | XP_001689480.1 |  |
| **RPS22** |  | PF3D7_1027200 |  | TGME49_110710 |  |  |  |  |  |  | |  |  |  |
| **RPS29** |  | PF3D7_1445300 |  | TGME49_110120 |  |  |  |  |  |  | |  |  |  |
| **RPS35** |  | PF3D7_1312300 |  | TGME49_003620 |  |  |  |  |  |  | |  |  |  |
|  | | | | | | | | | | | | | | |
| **RPL1** | PF3D7_1210000 | PF3D7_0710900 | TGME49_052270 | TGME49_044580 |  |  |  | BAC76180 | CMJ235C | EED86180 & ABK20741 |  | | XP_001701761 | EDO99703 |
| **RPL2** | CAA64579 | PF3D7_1132700 | AAD41134 | TGME49_025250 | YP_002990873 | XP_762672 | NP_852623 | BAC76234 | CMP227C | ABK20810 | YP_316609 | | NP_958369 | EDP04057 |
| **RPL3** | PF3D7_0918200 | PF3D7_1245400 | TGME49_043480 | TGME49_030050 |  |  |  | BAC76231 | CMF065C | ABK20807 |  | | XP_001700690 | XP_001689965 |
| **RPL4** | CAA64577 | PF3D7_0822000 | AAD41133 | TGME49_023660 | YP_002990874 | XP_762671 | NP_852622 | BAC76232 | CMQ421C | ABK20808 | EED93627 & ACI65024 | | XP_001697380 | XP_001697061 |
| **RPL5** | not found | not found | not found | not found |  |  |  | BAC76243 | NP_059372 | ABK20820 | YP_316605 | | NP_958373 |  |
| **RPL6** | CAA64586 | not found | AAD41140 | not found | YP_002990867 | XP_762681 | NP_852630 | BAC76245 | NP_059356 | ABK20822 | YP_316598 | | XP_001696829.1 |  |
| **RPL7/L12** | PF3D7_0524600 | PF3D7_0212200 | TGME49_089230 | TGME49_051950 |  |  |  | BAC76181 | CMR179C | ABK20742 | EED95349 | | XP_001693577 | XP_001701596 |
| **RPL9** | PF3D7_0310000 | PF3D7_1363300 | TGME49_214870 | not found |  |  |  |  |  |  |  | | XP_001694174.1 |  |
| **RPL10** | PF3D7_0413800 | not found | not found | not found |  |  |  | CMM011C |  | XP_002288521.1 |  | | XP_001696474 |  |
| **RPL11** | CAA64595 $ | PF3D7_1110600 # | AAD41147 | TGME49_054380 |  |  | NP_852638.2 | BAC76180 | CMT378C | ABK20740 | XP_002286368 | | XP_001702580 | XP_001697125 |
| **RPL13** | absent - but may be alternative splicing of PFB0645c N-term | PF3D7_0214200 | absent - but maybe alternative splicing of TGME49_025240 | TGME49_025240 |  |  |  | BAC76253 | CMH224C | ABK20830 | XP_001697603.1 | | XP_001696561 | XP_001697603 |
| **RPL14** | CAA64584 | PF3D7_0519100 | AAD41139 | TGME49_025250 | YP_002290870 | XP_762677 | NP_852628 | BAC76241 | NP_059373 | ABK20818 | YP_316604 | | NP_958372 |  |
| **RPL15** | PF3D7_1429100 | PF3D7_1429700 | TGME49_020300 | TGME49_053800 |  |  |  | CMQ292C | CMC132C | XM_002286840 |  | | XP_001698978 |  |
| **RPL16** | CAA64582 | PF3D7_1404400 | AAD41137 | TGME49_020150 | YP_002290871 | XP_ 762675 | NP_852626 | BAC76238 | NP_059374 | ABK20815 | YP_316612 | | NP_958371 |  |
| **RPL17** | PF3D7_0522500 | PF3D7_1431000 | TGME49_017590 | TGME49_097170 |  |  |  | CMM190C | CMT508C | EED89230 | EED92549 | | XP_001699718 | XP_001702909 |
| **RPL18** | PF3D7_0613400 | not found | TGME49_211870 | not found |  |  |  | BAC76246 | CMR285C | ABK20823 |  | | XP_001690256 |  |
| **RPL19** | not found | PF3D7_0610000 | not found | TGME49_113960 |  |  |  | BAC76184 | CME190C | EED86161 & ABK20716 | XP_001700862.1 | | XP_001693062 | XP_001700862 |
| **RPL20** | not found | PF3D7_1474100 **#** | not found | TGME49_093310 |  |  |  | BAC76219 | NP_059368 | ABK20737 | XP_001689789.1 | | NP_958363 | XP_001689789 |
| **RPL21** | PF3D7_0827500 | PF3D7_1422100 | TGME49_088610 # | TGME49_002350 |  |  |  | BAC76119 | CMS047C | ABK20791 & ABK20849 | XP_001694543.1 | | XP_001691294 | XP_001694543 |
| **RPL22** | PF3D7_1467400 | PF3D7_1010800 | TGME49_113550 # | TGME49_110490 |  |  |  | BAC76236 | CMS417C | ABK20813 |  | | Q84U21 |  |
| **RPL23** | CAA64578 | PF3D7_1239100 | not found | TGME49_260660 |  |  |  | BAC76233 | CMN112C | ABK20809 | XP_001697169.1 | | NP_958368 | EDP00424 |
| **RPL24** | PF3D7_1223900 # | PF3D7_0605000 # | TGME49_039720 # | TGME49_216010 # |  |  |  | BAC76242 | CMI055C | ABK20819 | EED88923 | | EDO99434 |  |
| **RPL27** | PF3D7_1034200 | PF3D7_0316100 | TGME49_093600 | TGME49_063110 |  |  |  | BAC76120 | CMP308C | ABK20792 & ABK20848 |  | | EDP05166 |  |
| **RPL28** | PF3D7_0503000 | PF3D7_1456600 | TGME49_009710 | TGME49_026280 |  |  |  | BAC76105 | CMJ238C | XP_002296284 |  | | EDP08523 |  |
| **RPL29** | PF3D7_1208000 | PF3D7_0315500 | TGME49_075330 # | TGME49_063550 |  |  |  | BAC76239 |  | ABK20816 | XP_001696595.1 | |  | EDP08572 |
| **RPL30** | not found | not found | not found | not found |  |  |  |  | CML149C |  | XP_001700671.1 | |  | XP_001700671 |
| **RPL31** | not found | not found | not found | not found |  |  |  | BAC76255 |  | ABK20832 |  | | XP_001696120.1 |  |
| **RPL32** | not found | not found | not found | not found |  |  |  | BAC76131 | CMA067C | ABK20789 & ABK20851 |  | | XP_001692618.1 |  |
| **RPL33** | PF3D7_0810100 | PF3D7_0210400 | TGME49_063020 | TGME49_108930 |  |  |  | BAC76276 | CMJ183C | EED86179 & ABK20748 | EED86095 | | XP_001698609 | XP_001698141 |
| **RPL34** | not found | not found | not found | not found |  |  |  | BAC76157 |  | ABK20749 & ABK20846 | EED95146 | | XP_001689581.1 |  |
| **RPL35** | PF3D7_0907800 | not found | TGME49_085530 | not found |  |  |  | BAC76218 | CMN012C | ABK20736 |  | | XP_001701250 |  |
| **RPL36** | CAA64589 | not found | AAD41142 | not found | ORF at 22463-22353 | XP_762684 | NP_852632 | BAC76249 | CMP301C | ABK20826 | XP_001694538.1 | | NP_958367 |  |
| **RPL37** |  | PF3D7_1123700 |  |  |  |  |  |  |  |  |  | |  |  |
| **RPL41** |  | PF3D7_0604200 |  |  |  |  |  |  |  |  |  | |  |  |
| **RPL43** |  | PF3D7_1010000 # |  |  |  |  |  |  |  |  |  | |  |  |
| **RPL46** |  | PF3D7_0626900 |  |  |  |  |  |  | CMH275C |  |  | |  |  |
| **RPL47** |  |  |  |  |  |  |  |  |  |  | EED92541 | |  |  |
| **RPL49** |  | PF3D7_1339500 |  |  |  |  |  |  |  |  |  | |  |  |

#, assigned by sequence similarity or by excluding other organellar counterpart, but targeting leader is non-obvious; $, contains an internal stop codon that may be suppressed. The L7-L12 dimer in eukaryotes is referred to as L8, but L7 and L12 are represented by a single gene in bacteria and organelles.

Text in **red** corresponds to mitochondrial-encoded proteins, text in **green** corresponds to plastid-encoded proteins. Only plastid-encoded ribosomal proteins are listed for the Apicomplexans *Babesia*, *Theileria* (Piroplasmida), and *Eimeria* (Coccidia).
